# Allatostatin C modulates nociception and immunity in *Drosophila*

**DOI:** 10.1038/s41598-018-25855-1

**Published:** 2018-05-14

**Authors:** Nathaniel D. Bachtel, Gary A. Hovsepian, Douglas F. Nixon, Ioannis Eleftherianos

**Affiliations:** 1 Department of Biological Sciences, George Washington University, Washington, DC 20052 USA; 2 Department of Microbiology, Immunology, and Tropical Medicine, George Washington University, Washington, DC 20037 USA

## Abstract

Bacterial induced inflammatory responses cause pain through direct activation of nociceptive neurons, and the ablation of these neurons leads to increased immune infiltration. In this study, we investigated nociceptive-immune interactions in *Drosophila* and the role these interactions play during pathogenic bacterial infection. After bacterial infection, we found robust upregulation of ligand-gated ion channels and allatostatin receptors involved in nociception, which potentially leads to hyperalgesia. We further found that *Allatostatin-C Receptor 2 (AstC-R2)* plays a crucial role in host survival during infection with the pathogenic bacterium *Photorhabdus luminescens*. Upon examination of immune signaling in *AstC-R2* deficient mutants, we demonstrated that *Allatostatin-C Receptor 2* specifically inhibits the Immune deficiency pathway, and knockdown of AstC*-R2* leads to overproduction of antimicrobial peptides related to this pathway and decreased host survival. This study provides mechanistic insights into the importance of microbe-nociceptor interactions during bacterial challenge. We posit that *Allatostatin C* is an immunosuppressive substance released by nociceptors or *Drosophila* hemocytes that dampens *IMD* signaling in order to either prevent immunopathology or to reduce unnecessary metabolic cost after microbial stimulation. *AstC-R2* also acts to dampen thermal nociception in the absence of infection, suggesting an intrinsic neuronal role in mediating these processes during homeostatic conditions. Further examination into the signaling mechanisms by which *Allatostatin-C* alters immunity and nociception in *Drosophila* may reveal conserved pathways which can be utilized towards therapeutically targeting inflammatory pain and chronic inflammation.

## Introduction

In recent years there has been a growing body of research investigating the role of the inflammatory response in causing pain during bacterial infections including the discovery of interactions between bacteria, pain-sensing neurons called nociceptors, primary sensory afferents and the innate immune system^1^. Upon activation by proinflammatory cytokines, bacterial lipopolysaccharides, flagella or a-hemolysin, specific ligand-gated ion channels (TRPA1, FRPR1, ADAM10) open, resulting in an action potential propagating throughout these neurons^2^. Once the synapse is reached, these nociceptors release various immunomodulatory neuropeptides into the proximal vicinity including somatostatin, substance-P, CGRP and VIP^3,4^. These neuropeptides have been shown to have a bimodal effect by altering further nociception, as well as having varied effects on inflammation. In fact, ablation of subcutaneous nociceptors has been shown to increase immune infiltration in mice during *Staphylococcus aureus* infection whereas ablation near respiratory airways has been shown to reduce inflammation in a murine asthma model^1,5^. Due to bacteria being able to directly activate these nociceptive neurons and many products of these neurons altering systemic immunity, the question then arises as to whether the ability of bacteria to activate nociceptive neurons is beneficial or detrimental to the host^6^.

The common fruit fly, *Drosophila melanogaster*, provides an excellent opportunity to investigate these interactions for numerous reasons. *Drosophila* is a well-established model for probing questions relating to the innate immune response during microbial infection^7–14^. Moreover, *Drosophila* possesses primitive nociceptive neurons that are able to respond to noxious temperatures, mechanical stimuli, as well as harmful chemicals via sensory-gated ion channels^15–17^. Interestingly, these neurons can also be activated by the proinflammatory cytokine, *Eiger*, and bacterially derived LPS, suggesting a greater degree of functional homology to mammalian systems than previously realized^18,19^.

Activation of nociceptive neurons in *Drosophila* leads to an aversion to these noxious stimuli primarily through avoidance behaviors^20–24^. Beyond this behavioral output, nociceptive neurons in *Drosophila* are also linked with immune cell differentiation. RNAi knockdown of two genes crucial for nociceptor formation, *painless* and *piezo*, has been shown to alter lamellocyte differentiation during parasitoid wasp infection, demonstrating that nociceptor activation and cell-based immunity are linked in this invertebrate organism^25^.

The aim of the current study was to characterize a panel of known nociceptive (*TRPA1*, *ppk*, *AstA-R1*, *AstC-R1*, *AstC-R2)* genes in *Drosophila* (Table 1), and to determine if any of these genes impacted survivorship or immune function during bacterial infection. For this study, loss-of-function fly mutants for each gene were generated and injected with either a non-pathogenic strain of *E*. *coli* or the insect-pathogenic bacterium *P*. *luminecens*. Following infection, noxious heat threshold, survival, immune gene expression, and bacterial load in each mutant were analyzed. Results showed that genes coding for *TRPA1*, *ppk*, *AstA-R1*, *AstC-R1*, and *AstC-R2* are upregulated during bacterial infection and this upregulation may lead to hyperalgesia. Interestingly, RNAi knockdown of *AstC-R2*, a receptor for a neuropeptide hormone released from nociceptors that is homologous to mammalian somatostatin (Supplementary Fig. 1), led to a significant decrease in fly survival during *P*. *luminecens* infection. Further characterization of this gene’s role during infection with the pathogen suggests an immune deficiency (*IMD*)-specific suppressive mechanism of action that, when removed, leads to an over-exuberant inflammatory response and subsequently premature death. Our findings indicate that nociceptive-related genes are upregulated during infection of *Drosophila* with insect pathogenic bacteria and that neuropeptides released from nociceptive neurons play a significant role in the regulation of the host antibacterial immune response.

**Table 1:** Immune and nociceptive related genes in *Drosophila melanogaster* probed in this study with their corresponding functions and human homologs.

Gene in *Drosophila*	Function	Human Ortholog
*Allatostatin-A Receptor 1*	Allatostatin A binding Neuropeptide receptor activity GPCR activity	*Galanin receptor 2*
*Allatostatin-C Receptor 1*	Allatostatin C binding Neuropeptide receptor activity GPCR activity	*Somatostatin receptor 2*
*Allatostatin-C Receptor 2*	Allatostatin C binding Neuropeptide receptor activity GPCR activity	*Somatostatin receptor 2/4*
*Transient Receptor Potential Cation Channel A1*	Ligand gated cation channel activity Temperature gated channel activity	*Transient Receptor Potential Cation Channel A1*
*Pickpocket*	Epithelial sodium channel Noxious temperature and acid sensing activity	*Acid sensing ion channel subunit 2*
*Cecropin A1*	Antimicrobial peptide activity against gram-negative bacterium	*N/A*
*Drosomycin*	Antimicrobial peptide activity against gram-positive bacterium	*N/A*
*Eiger*	Tumor necrosis factor binding activity Inflammatory pain activity	*TNF superfamily member 13b*

## Results

### Nociceptive-related genes are differentially upregulated in response to *E*. *coli* or *P*. *luminescens*

Due to prior studies showing allodynia following UV radiation in *Drosophila*, we sought to determine whether bacterial infection could also alter sensitivity to painful stimuli^18^. Hyperalgesia is the result of increased transcription and subsequent translation of ligand gated ion channels in nociceptive neurons^26^. Therefore, we determined whether nociceptive-related genes, including *TRPA1*, *ppk*, *AstA-R1*, *AstC-R1*, and *AstC-R2*, were upregulated during bacterial infection and whether the expression of these genes differed upon infection. We injected 7–10 day old wild-type flies with *E*. *coli*, *P*. *luminescens*, or PBS as a septic injury negative control, and monitored their transcript level activity over the course of the infection.

**Figure 1:**
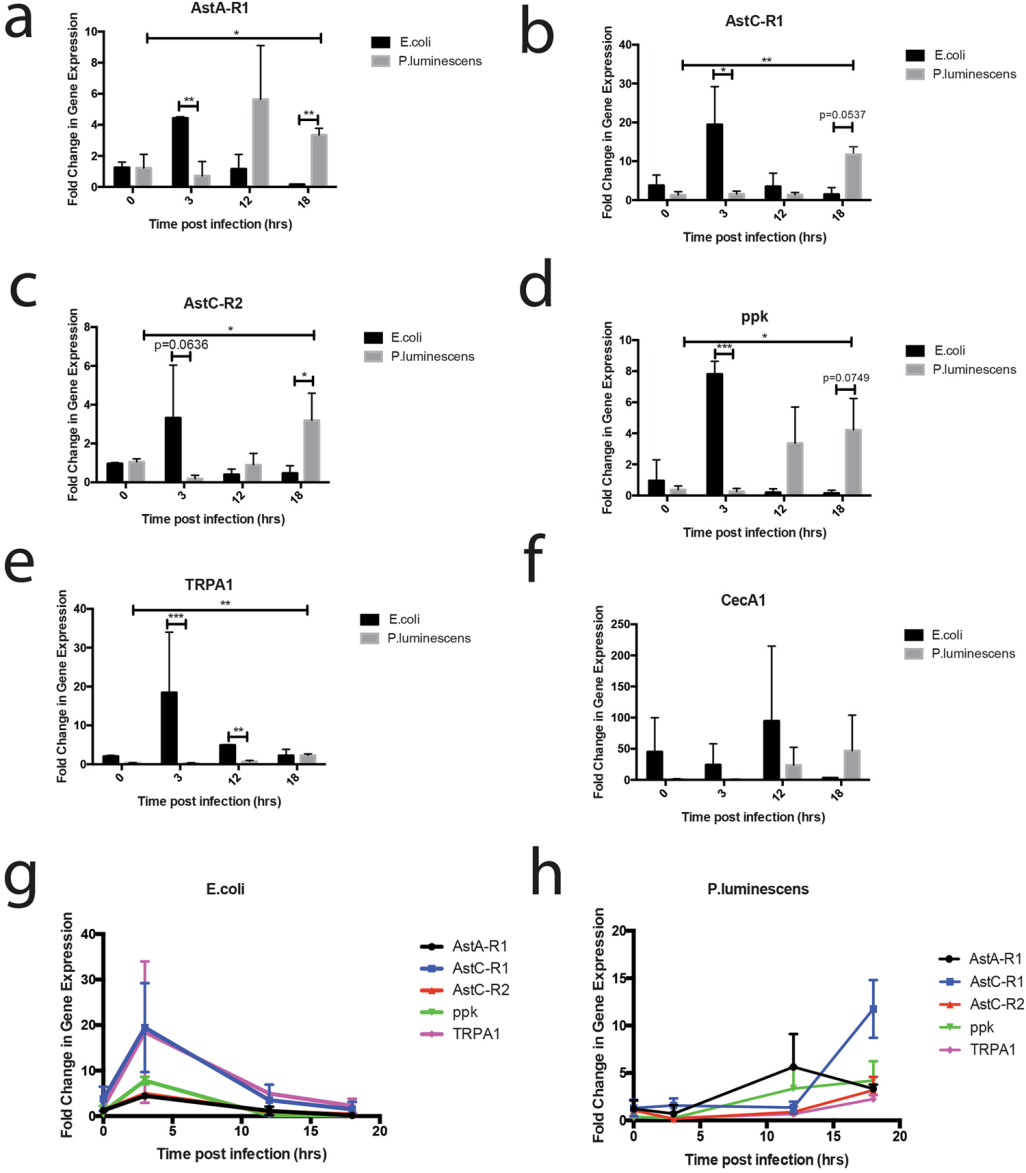
Nociceptive gene expression in *Drosophila* differs temporally during bacterial infection. Expression of (**a**) *AstA-R1* (**b**) *AstC-R1* (**c**) *AstC-R2* (**d**) *ppk* (**e**) *TRPA1* and (**f**) *Cecropin A1* (CecA1) in *Oregon* flies responding to non-pathogenic *E*. *coli* or pathogenic *P*. *luminescens* bacteria at 0, 3, 12, and 18 hours post infection. (**g**)Upon infection with *E*. *coli*, all pain-related genes are upregulated at 3 hours and their mRNA levels decrease to basal levels by 12 hours. (**h**) Upon *P*. *luminescens* infection nociceptive gene transcript levels increase to a peak at either at 12 or 18 hours post infection. Differences in gene expression profiles were analyzed for statistical significance using a student’s t-test (n = 3–4 groups of 10 flies per time point, *p < 0.05, **p < 0.01, ***p < 0.0001).

Our results demonstrate that all five nociceptive genes were upregulated during infection with *E*. *coli* or *P*. *luminescens* as compared to PBS. We found that nociceptive gene upregulation temporally differed between infections (Fig. 1). *AstA-R1* expression was significantly upregulated (p = 0.0064) between 0 and 3 hours post infection with *E*. *coli*, before decreasing between 3 and 12 hours post infection (Fig. 1a). For *P*. *luminescens*, *AstA-R1* expression peaked later during the infection, upregulated between 3 and 12 hours and 3 and 18 hours post infection (p = 0.0335 and p = 0.0063, respectively) (Fig. 1a). Transcript levels were higher in the *E*. *coli* infected flies at 3 hours, in contrast to *P*. *luminescens* infected flies, which peaked at 18 hours. A similar pattern was seen for transcript levels of *AstC-R1*, *AstC-R2*, *TRPA1*, and *ppk* (Fig. 1b–h).

### Expression of nociception-related genes better correlates with bacterial load than with immune induction

**Figure 2:**
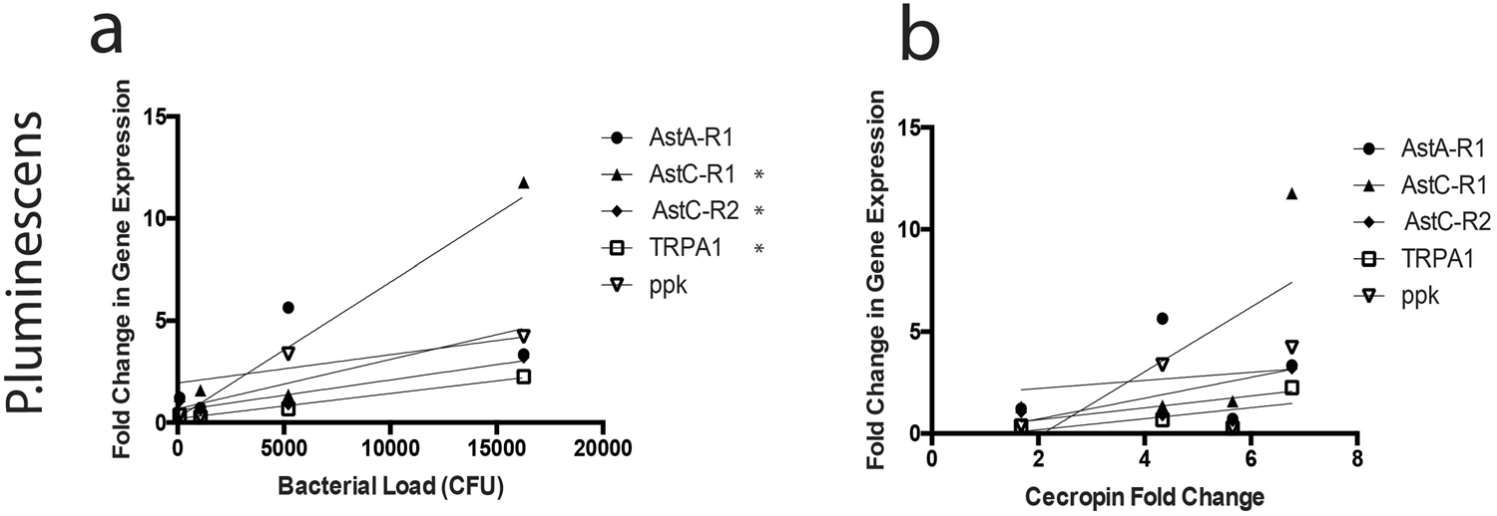
Allatostatin-C receptors and TRPA1 expression in *Drosophila* significantly correlates with bacterial load during *P*. *luminescens* infection. Correlation and linear regression lines for nociceptive gene expression in *w*^1118^ flies over time plotted against (**a**) bacterial load and (**b**) *Cecropin A1* expression following *P*. *luminescens* infection. Bacterial load significantly correlates with *TRPA1*, *AstC-R1*, and *AstC-R2* expression upon infection with *P*. *luminescens* via two-tailed linear regression analysis (n = 3–4 groups of 10 flies per time point, *p < 0.05).

Upon attempting to correlate nociceptive gene transcript levels with bacterial load or immune activation as measured by the induction of an antimicrobial peptide gene readout of the IMD pathway, *Cecropin A1*, it was surprising to find that *TRPA1*, *AstC-R1*, and *AstC-R2* expression significantly correlated with bacterial load (p = 0.011, p = 0.047, p = 0.0246 respectively) (Fig. 2a) but not *Cecropin A1* transcript levels (p = 0.358, p = 0.329, p = 0.457 respectively) (Fig. 2b) during *P*. *luminescens* infection, suggesting bacteria may play an active role in their induction. The same analysis with *E*. *coli* demonstrated that nociceptive gene transcript levels better correlated with bacterial load (Supplementary Fig. 2a) than immune activation (Supplementary Fig. 2b), yet neither of these correlations were statistically significant (p > 0.05).

### *Drosophila IMD* and *TRPA1* RNAi mutants display hypoalgesia whereas *AstC-R1* and *AstC-R2* mutants display hyperalgesia

**Figure 3:**
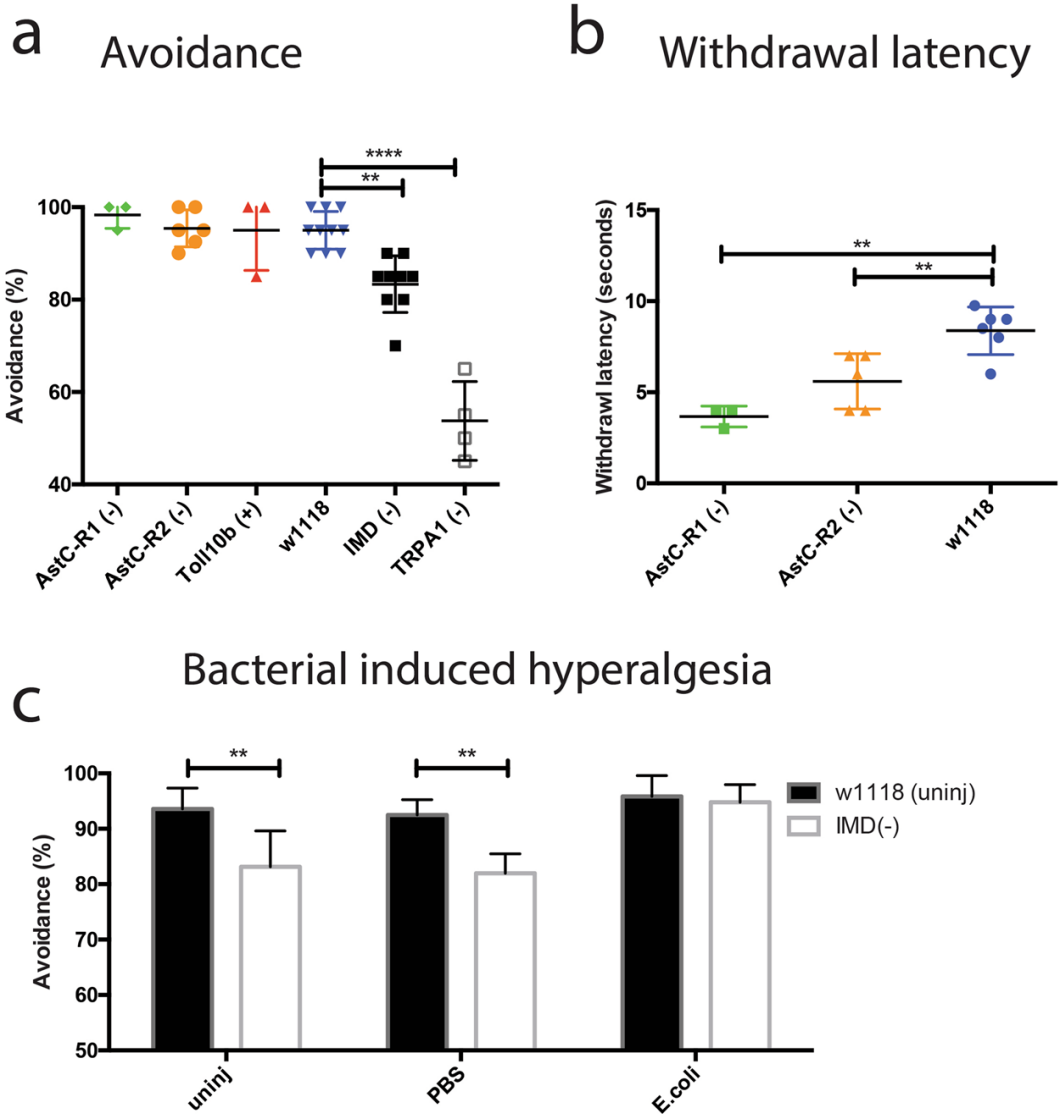
Nociceptive and immune *Drosophila* mutants display alterations to pain sensing which can be manipulated via bacterial challenge. (**a**) Noxious heat threshold of *Drosophila* RNAi mutants for nociceptive and immune related genes. *IMD* RNAi mutants as well as *TRPA1* mutants display a hypoalgesia whereas the *Toll 10b* and *AstC-R1* and *AstC-R2* RNAi mutants do not display a reduced noxious heat sensing capacity. (**b**) *AstC-R2* and *AstC-R1* RNAi mutants display reduced withdrawal latency. (**c**) *IMD* RNAi mutants display hyperalgesia upon infection with *E*. *coli* but not upon PBS injection or in uninjected controls. RNAi mutants were generated by crossing UAS-RNAi lines with an Actin5c Gal4 driver in order to knock the gene of interest down ubiquitously. (−) Indicates the use of an RNAi line whereas (+) indicates a line that constitutively expresses the gene of interest. Differences in noxious-related behaviors between *Drosophila* RNAi mutants were analyzed for statistical significance using a student’s t-test (n = 3–9 groups of 20 female flies, *p < 0.05, **p < 0.01, ***p < 0.0001).

To better understand as to whether pain sensitization was linked to the immune response or bacterial injection, we tested various immune and nociceptor *Drosophila* mutants using a noxious heat escape assay to measure hypoalgesia and a withdrawal latency assay to measure hyperalgesia. The noxious heat threshold of *w*^1118^ flies was a mean of 94% for the heat escape assay and these flies had a mean withdrawal latency of 7.7 seconds (Fig. 3a,b).

We determined that *IMD* knockdown mutants had a significantly increased pain threshold compared to wild-type flies (mean = 83% vs 95%, p = 0.0003) (Fig. 3a). We found no significant changes in the pain threshold of *Toll 10b* flies, a strain that constitutively expresses the Toll pathway (p = 0.99). Further, we found that the pain threshold of *IMD* knockdown mutants was significantly decreased by injection of *E*. *coli* (83% vs 95%, p = 0.003) (Fig. 3c), suggesting that *IMD* knockdown is not sufficient to abolish inflammatory pain in *Drosophila*.

*TRPA1* knockdown mutants displayed a significantly increased pain threshold as compared to wild-type flies (mean = 54% vs 94%, p < 0.0001) (Fig. 3a). This result is consistent with previous studies demonstrating the importance of *TRPA1* in sensing noxious temperatures^26^. We found a trend towards a decrease in the pain threshold of *AstC-R1* knockdown mutants using the heat escape assay (mean = 98% vs 95%, p = 0.219) and a significant decrease in withdrawal latency (mean = 8.4s vs 3.7s, p = 0.006). Similarly, we saw a trend in the pain threshold of *AstC-R2* knockdown mutants and wild-type flies (mean = 96% vs 95%, p = 0.86), while there was a significant decrease in their withdrawal latency (mean = 8.4s vs 5.6s, p = 0.009) (Fig. 3a,b).

### RNAi knockdown of *AstC-R2* increases susceptibility to *P*. *luminescens* infection

**Figure 4:**
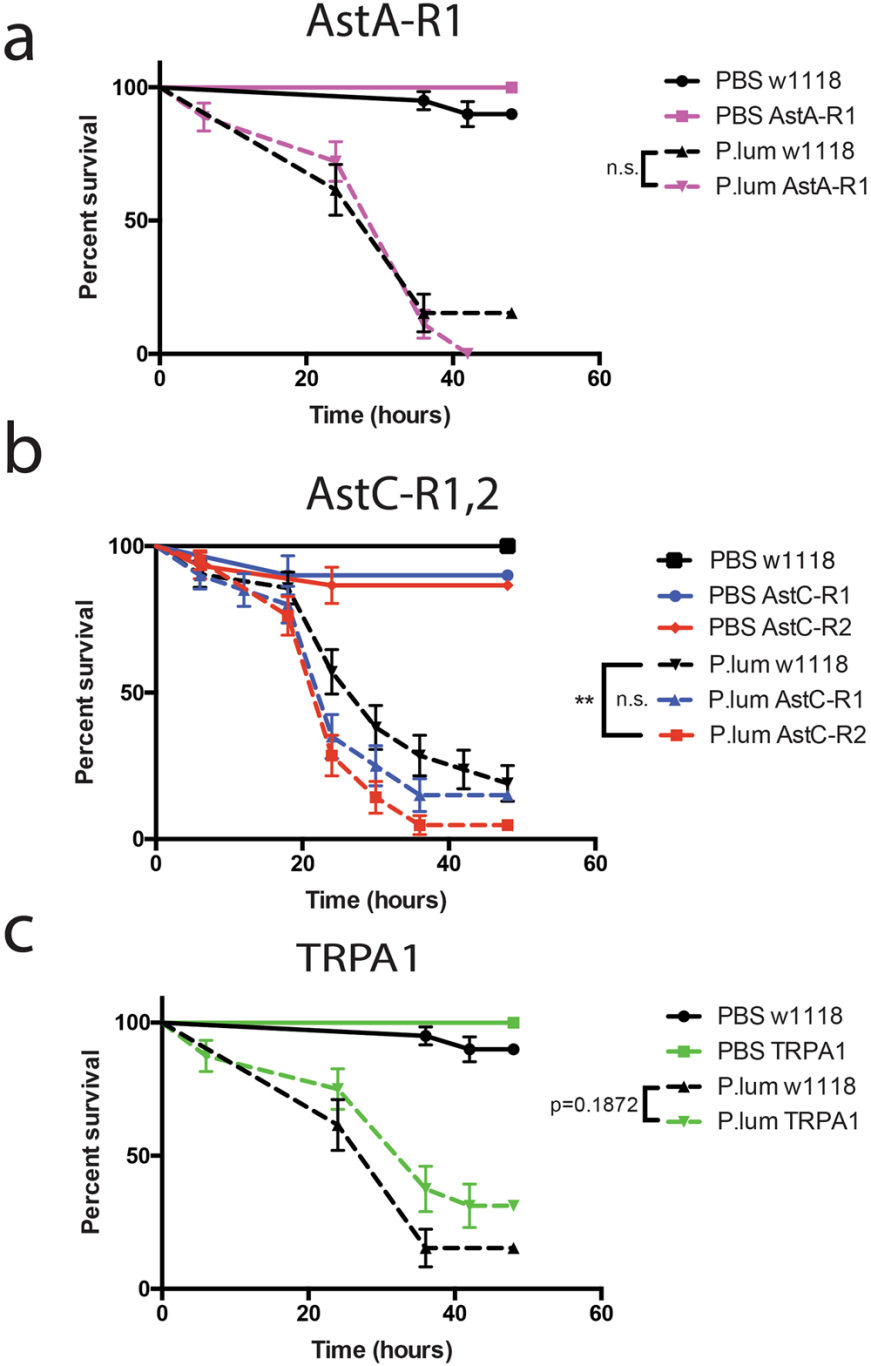
Allatostatin-C Receptor *Drosophila* mutants show increased susceptibility to *P*. *luminescens* infection. Survival curves of RNAi mutant flies for (**a**) *AstA-R1* (**b**) *AstC-R1* and *AstC-R2* and (**c**) *TRPA1* post injection with PBS, *P*. *luminescens*, and *E*. *coli*. Although neither *AstA-R1* and *TRPA1* RNAi mutants displayed a reduced survival during *P*. *luminescens* infection, *AstC-R1* and *AstC-R2* RNAi flies succumbed faster than their controls with *AstC-R2* RNAi individuals showing significantly increased sensitivity to the pathogen (p < 0.01). RNAi mutants were generated by crossing UAS-RNAi lines with an Actin5c Gal4 driver in order to knock the gene of interest down ubiquitously. Survival curves were analyzed using survival curve analysis in GraphPad Prism software (n = 3 groups of 20 flies, **p < 0.01).

Bacteria can directly activate nociceptive neurons in *Drosophila* and the ablation of these neurons in mice alters immune infiltration^1,18^. We thus determined whether nociception-related genes are beneficial or detrimental to the host upon bacterial infection. To test this, we generated RNAi knockdown mutants for each nociception-related gene and measured the survival of flies over the course of infection with *P*. *luminescens*. Our results show that knockdown of nociception-related genes had varying effects on the survival of the flies during bacterial infection (Fig. 4).

There was no significant change in survival of *AstA-R1* knockdown mutants as compared to wild-type flies during *P*. *luminescens* infection (p = 0.543) (Fig. 4a). In contrast, knockdown of *AstC-R1* trended towards a decrease in host survival whereas knockdown of *AstC-R2* significantly reduced host survival (p = 0.0056) (Fig. 4b). Although knockdown of *AstC-R2* reduced survival during *P*. *luminescens* infection, it was not sufficient to change susceptibility to infection with a non-pathogenic strain of *E*. *coli (*p = 0.15) (Supplementary Fig. 3a). Finally, knockdown of *TRPA1*, a known point of interaction between bacteria and host-nociceptor in *Drosophila*, trended towards an increase in survival as compared to wild-type flies during *P*. *luminescens* infection (p = 0.1872) (Fig. 4c).

### RNAi knockdown of *AstC-R2* hyperactivates the IMD pathway without reducing bacterial load

**Figure 5:**
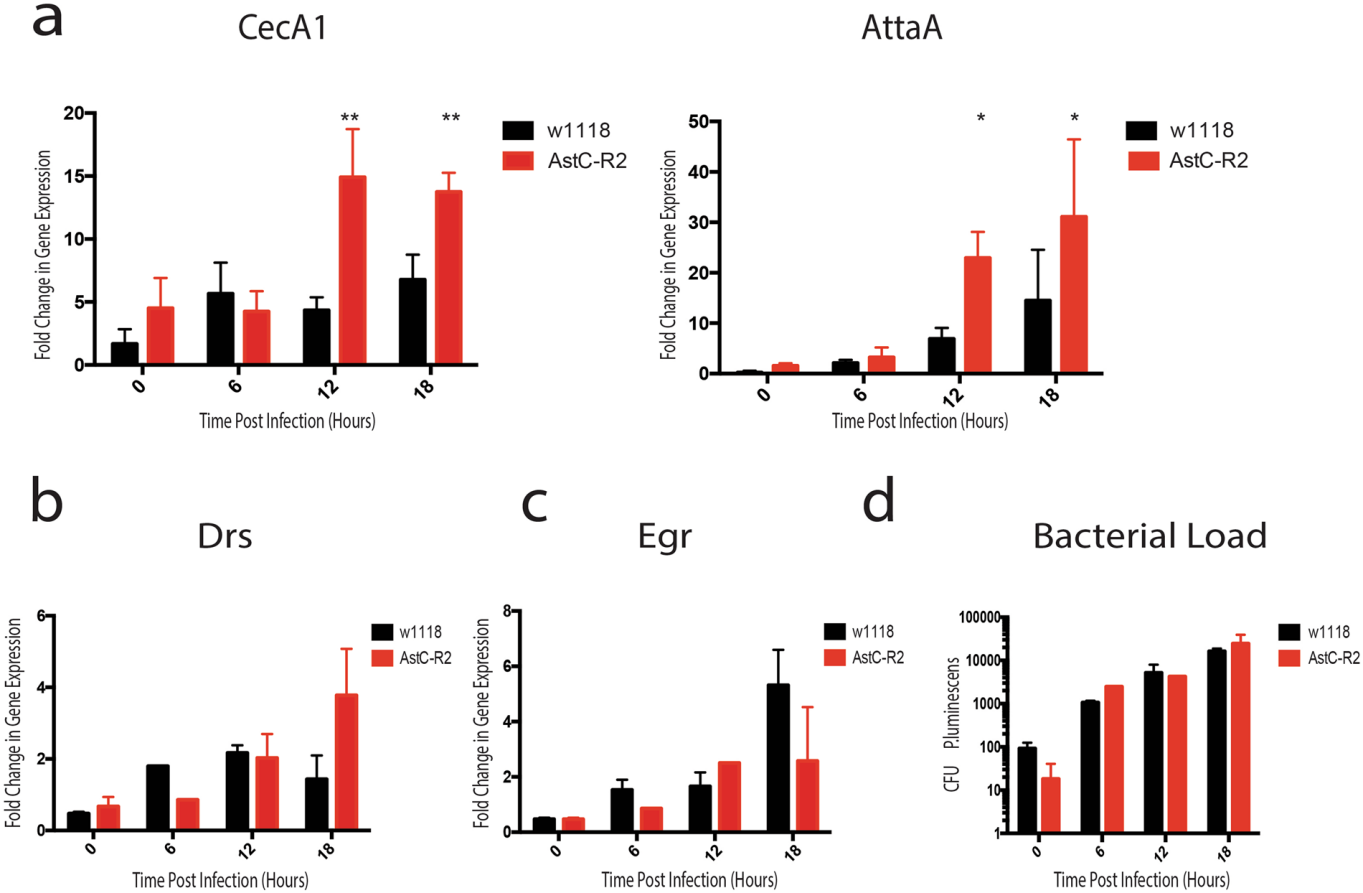
AstC-R2 RNAi *Drosophila* mutants display hyperactive IMD signaling without altered bacterial load. Immune gene expression of *AstC-R2* RNAi mutant and background control flies following infection with *P*. *luminescens* bacteria. *AstC-R2* RNAi mutant flies display (**a**) upregulation of the antimicrobial peptide-encoding genes *Cecropin A1* (CecA1) and *Attacin A* (AttaA) which are controlled by the IMD pathway with (**b**) a modest increase in expression of the antimicrobial peptide-encoding gene *Drosomycin* (Drs) which is regulated by Toll signaling, and (**c**) decrease in *Eiger* (egr) expression. (**d**) *AstC-R2* flies demonstrate no significant decrease in bacterial load over the course of *P*. *luminescens* infection. RNAi mutants were generated by crossing UAS-RNAi lines with an Actin5c Gal4 driver in order to knock the gene of interest down ubiquitously. Differences in gene expression profiles were analyzed for statistical significance using a student’s t-test (n = 2–5 groups of 10 flies per time point per genotype, *p < 0.05, **p < 0.01).

Due to significant decrease in survival of *AstC-R2* knockdown flies upon infection with *P*. *luminescens*, we sought to determine whether alterations in NF-kB immune pathway activation and bacterial load in these flies could explain this effect. We found a statistically significant hyperactivation of *Cecropin A1* and *Attacin A*, two antimicrobial peptide readouts of the *IMD* pathway as compared to wild-type flies (Fig. 5a) at 12 and 18 hours post infection with *P*. *luminescens (CecA1:* p = 0.0029, p = 0.0040, *AttA:* p = 0.048, p = 0.014 respectively). We saw a similar hyperactivation in *IMD* signaling after infection by the non-pathogen, *E*. *coli* as well (Supplementary Fig. 3b). However, transcript levels of *Cecropin A1* did not differ between *AstC-*R2 RNAi knockdown flies and wild-type flies in the absence of bacterial injection (Supplementary Fig. 4). Additionally, we observed a modest, yet non-significant increased activation of the *Toll* pathway as measured by expression of *Drosomycin* at 18 hours post infection (p = 0.15, Fig. 5b) and a slight decrease in Jak-Stat activation as measured by transcriptional expression of *Eiger* at 18 hours post infection (p = 0.09, Fig. 5c). Surprisingly, despite this robust increase in IMD signaling, we observed no statistically significant differences in the bacterial load in the *AstC-R2* knockdown flies upon *P*. *luminescens* infection at any timepoint (p > 0.05, Fig. 5d).

## Discussion

During bacterial challenge, the host immune response must be mounted in a tightly regulated and quantitatively precise manner. Overproduction of immune effectors results in immune-related pathophysiology, tissue damage, and metabolic cost whereas under-production of these effectors may permit bacterial expansion and subsequently bacterially derived damage^27–30^. Recent studies have shown that bacteria can directly interact with nociceptive neurons, and that ablation of these neurons leads to increased lymph drainage during *S*. *aureus* infection most likely by suppressing immunomodulatory neuropeptide release. Thus, bacterial activation of nociceptive neurons may be a novel mechanism of immune control. This study represents the first attempt to characterize bacterially induced hyperalgesia and the effects of genes related to this process on host immunity in *Drosophila melanogaster*. Our study provides support for a newly emerging idea that nociceptive neurons may be crucial to mounting an appropriate immune response during these infections^1,2,22^.

We investigated the gene kinetics, effect on noxious behavior, and immune consequences of nociceptive gene activation during microbial challenge. We found a robust upregulation of ligand gated ion channels (*TRPA1* and *ppk*) and Allatostatin receptors (*AstC-R1*, *AstC-R2*, *AstA-R1*) upon microbial challenge, the homologs of both of which have been associated with hyperalgesia in mammalian systems^31–37^. We found that nociceptive gene activation differed temporally upon infection with *E*. *coli* as compared to pathogenic *P*. *luminescens*, and that bacterial load better correlated with nociceptive gene activation than immune activation (as measured by the IMD antimicrobial peptide encoding gene, *Cecropin A1)*. Importantly, this correlation supports a recent paper demonstrating that *S*. *aureus* bacterial load better correlates with hyperalgesia than paw swelling (immune infiltration) in mice^1^.

To determine whether the upregulation of these nociception-related genes contributed to hyperalgesia, we generated immune and nociceptive knockdown fly mutants for the genes upregulated, and measured changes to noxious heat sensitization. Upon examining alterations to this behavior, we found that *AstC-R1* and *AstC-R2* RNAi mutants displayed hyperalgesia whereas *IMD* and *TRPA1* knockdown mutants showed robust hypoalgesia. These results are in agreement with previous studies demonstrating the importance of *TRPA1* in noxious heat sensation^38^. To determine whether we could raise the noxious heat sensitivity of *IMD* mutants back to wild-type levels by infection with a bacterium, we infected *IMD* knockdown flies with a non-pathogenic strain of *E*. *coli* and found that these mutants displayed hyperalgesia, suggesting IMD activation contributes to, but is not necessary for hyperalgesia during bacterial infections. These results implicate NF-kB activation as a conserved mechanism of hyperalgesia in arthropod and mammalian lineages with the additional hyperalgesia seen upon infection of *IMD* knockdown mutants being attributed to Toll signaling or direct bacterial activation^39–41^. Indeed, previous studies have found that a transcription factor downstream of *IMD* activation, *Relish*, alters thermal nociception as well^17,22^.

Due to bacteria being able to potentially manipulate the expression of nociceptive genes in their favor, we were curious as to whether any of the nociception-related genes tested played a beneficial or detrimental role to the host during microbial challenge. To test this, we silenced each nociception-related gene ubiquitously in flies and measured their survival upon injection with the insect pathogen *P*. *luminescens*. We found a trend towards decreased survival of *AstC-R1* knockdown flies and a significant decrease in survival upon knockdown of *AstC-R2*, suggesting a potential role for *Allatostatin-C* in modulating host immune processes during bacterial infection. However, when infecting *AstC-R2* knockdown flies with the non-pathogen *E*. *coli*, we observed no decreased survival over 48 hours hours as compared to wild-type flies suggesting that this effect alone is not sufficient to cause death.

The mammalian homolog of *Allatostatin* is *Somatostatin*^42–44^, which has documented effects in reducing systemic inflammation in mammalian systems, and thus we examined whether knockdown of *AstC-R2* leads to alterations in immune signaling that could contribute to the decreased survival^4,45,46^. We observed a robust over induction of IMD signaling with a modest, but non-significant increase in *Toll* and decrease in *Eiger* as compared to wild-type flies, suggesting that *AstC-R2* reduced IMD signaling independently of the Toll or Jak-Stat pathways respectively. Despite the robust upregulation of the IMD pathway, we observed no changes in bacterial load during *P*. *luminescens* infection of *AstC-R2* knockdown flies as compared to wild-type controls. These results suggest that antimicrobial peptides related to this pathway are ineffective at controlling this pathogen. Indeed, recent reports have shown that an antimicrobial peptide-resistant sub-population of *P*. *luminescens* is responsible for the majority of the virulence during insect infection, and that *P*. *luminescens* is able to employ proteases that specifically degrade antimicrobial peptides, rendering them post-translationally ineffective^47,48^.

By knocking down a receptor for *Allatostatin C*, which has dual role in inhibiting heat-driven nociception as well as inhibiting the IMD pathway during bacterial challenge, we observed hyperactivation of this immune pathway, hyperalgesia, and reduced survival upon challenge with *P*. *luminescens*. The hyperalgesia seen in *AstC-R1* and *AstC-*R2 RNAi knockdown flies in the absence of bacterial challenge most likely is not due to dysregulation of the IMD pathway because we observed similar basal transcript levels of *Cecropin A1* in *AstC-R2* knockdown mutants as compared to wild-type flies (Supplementary Fig. 3). Indeed, *AstC-R1* and *AstC-R2* also share structural homology with mammalian opioid receptors^22^. However, the reduced survival in *AstC-R2* knockdown flies may be explained either directly or indirectly by over activated IMD signaling and *AstC-R2-IMD* double knockdown mutants will be needed in order to confirm this hypothesis. Remarkably, our results recapitulate many of the findings found in a seminal study investigating the importance of somatostatin receptor 4 in the modulation of hyperalgesia and inflammation^49^. Therefore, *Drosophila* AstC-R2 may be more functionally similar to mammalian SSTR4 than previously perceived.

Due to the transcriptional upregulation of *AstC-R1* and *AstC-R2* during infection, it is likely that this upregulation reflects one mechanism of the host fine-tuning the immune response to prevent immune related damage from occurring as well as mediating avoidance behaviors while in a compromised state. Somatostatin regulatory circuits have been documented at sites of chronic inflammation where they have important roles in inhibiting pro-inflammatory cytokine production by macrophages and T-cells yet found processes have not been previously described in *Drosophila*^50–53^. Interestingly, another neuropeptide that acts as a crucial component of this circuit by inhibiting somatostatin release is substance P, an additional molecule released from nociceptive neurons^54,55^. Thus, immune manipulation during microbial challenge by nociceptive neurons is likely to be a well-orchestrated process that amplifies or suppresses pro-inflammatory cytokine production in a way to best ensure host survival.

**Figure 6:**
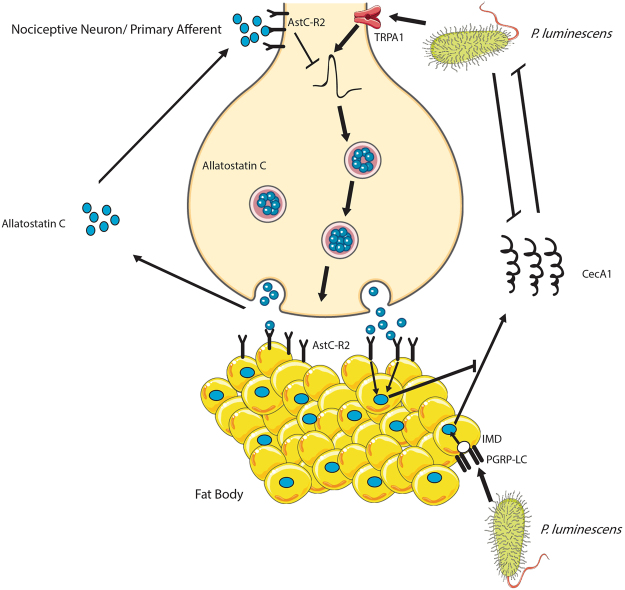
Potential role for AstC-R2 in nociceptor-bacterial-immune interactions in *Drosophila*. Upon bacterial infection by a Gram-negative pathogen, the IMD pathway is activated by DAP-type peptidoglycan, NF- κB is activated and translocates to the nucleus, and transcription of effector antimicrobial peptide-encoding genes related to this pathway (*CecA1*) occurs. Simultaneously, TRPA1 channels open by direct interaction with bacterial LPS or N-formyl peptides leading to nociceptive neuron firing and the subsequent release of Allatostatin C. In turn, Allatostatin C inhibits the IMD pathway as well as heat driven nociception through binding to AstC-R2 on the fat-body cells and nociceptors respectively, thus completing a negative regulatory circuit controlling IMD activation. Figure was modified from images from Servier Medical Art, licensed under a Creative Common Attribution 3.0 Generic License. http://smart.servier.com/.

Our results imply that nociceptor-immune interactions during microbial infection in *Drosophila* may be more similar to mammalian systems than previously conceived (Fig. 6). This idea is supported by recent findings demonstrating that nociceptive neurons in flies are sensitive the proinflammatory cytokine *Eiger*, as well as bacterially derived lipopolysaccharides^18,19^. *Drosophila* also possesses homologous genes for other immuno-modulatory substances released from nociceptors including *substance P*, *CGRP* and *VIP* (*DTK*, *DH31*, and *Pdf* respectively), yet their roles in pain sensation and immunity have not been characterized^56–58^. Due to the wealth of transgenic lines available, quick developmental cycle and cheap cost of maintenance, *Drosophila* could prove to be a valuable tool in deciphering nociceptor-innate immune interactions in the future. Further studies into the interface of pain, immunity, and microbial challenge hold large promise for innovative treatments for inflammatory pain, auto-immune conditions, as well as potential explanations for host-tolerance of the gut microbiota.

## Materials and Methods

### Bacterial preparation

Bacteria were stored as 20% glycerol stocks at −80 **°**C before use. Bacteria were thawed and then grown in 10 mL of Luria-Bertani broth. *Escherichia coli* and *Photorhabdus luminescens* were grown at 30 **°**C for 18 hours or 22 hours, respectively. After incubation, the bacterial solutions were pelleted by centrifugation for 5 minutes at 4 **°**C at 3,000 rpm. The pellets were washed twice before resuspension in PBS. Concentrations were adjusted using a Nanodrop 2000 spectrophotometer with an absorbance at 600 nm denoting the respective concentrations. *E*. *coli* was used at an optical density (OD) of 0.015, while *P*. *luminescens* was used at an OD of 0.10. This OD corresponds to between 300–1000 colony forming units (cfu) of each bacterium.

### *Drosophila* mutants and crosses

The following *Drosophila* strains were obtained from the Vienna *Drosophila* Resource Center (Vienna, Austria) or Exelixis at Harvard Medical School (Cambridge and Massachusetts*)*; *Oregon* and *w*^*1118*^, *AstA-R1:* v3400 and v3399, *AstC-R1*: v13560 and v110739, *AstC-R2:* v50000 and v106146, *TRPA1*: v37249 and v37250, *ppk:* v108683, *Toll 10b* and *IMD* (*−*), *Actin5c-Gal4*. UAS-RNAi *Drosophila* lines were crossed with the Actin5C Gal4 driver in order to ubiquitously silence the gene of interest in the resulting progeny. Reduced transcriptional activity of each gene silenced via-RNAi was confirmed via quantitative RT-PCR (qRT-PCR) (Supplementary Fig. 5).

### Fly injection

*Drosophila melanogaster* flies aged from 7–10 days were anesthetized using carbon dioxide. 10–12 flies were then injected with 18.4 nl of the standardized bacterial solution using a Nanoject microinjector fitted with capillary needles. PBS was used as a septic injury negative control for all experiments. Flies were collected after injection by freezing at −80 **°**C.

### Gene expression and bacterial load determination

RNA extractions were carried out using PrepEase RNA Spin Kits *(*USB*)* or Trizol Reagent (Thermofisher) and eluted using molecular grade H_2_O. cDNA syntheses were carried out using 300 ng of RNA with High Capacity Reverse Transcription cDNA Synthesis Kit (Applied Biosystems). The cDNA was then diluted 1/10 times before proceeding to qRT-PCR analysis. All qRT-PCR assays were carried out using CFX96 Real-Time System (Bio-Rad). 1 µl of cDNA was used per reaction using gene-specific primers (Table 2) (Eurofins MWG Operon) and SYBR Green Supermix (Bio-Rad). Ct values were analyzed using the Delta Ct method using *RpL32* as the control gene, and PBS as the control treatment. Bacterial load was calculated using this method in conjunction with measuring the expression of 16S rRNA.

**Table 2:** Primers used in quantitative RT-PCR analysis of immune and nociception related genes in *Drosophila melanogaster*.

Gene	Forward (5′-3′)	Reverse (5′-3′)
*AstA-R1*	CACGGCTACCGATTACGTGC	AAGGACATCAGCACCAGCGT
*AstC-R1*	GGTCCCAAACCAGGAACGAA	GAAATCCAGTGAGGGAGCCA
*AstC-R2*	CTGCCCGCAAGGATGTGA	TGTCGTCGTCGTTGTAGTGG
*TRPA1*	GACTTCGGGCGACAAGGAGA	CTCGCCCCACTGGAAGAAGA
*ppk*	AGCACGACCATTCACGGCATAC	CCAAAGTTCACTCACTGGGCATC
*Cecropin A1*	TCTTCGTTTTCGTCGCTCTC	CTTGTTGAGCGATTCCCAGT
*Drosomycin*	GACTTGTTCGCCCTCTTCG	CTTGCACACACGACGACAG
*Eiger*	AGCTGATCCCCCTGGTTTTG	GCCAGATCGTTAGTGCGAGA
*Attacin A*	CAATGGCAGACAATCTGG	ATTCCTGGGAAGTTGCTGTG
*rp49*	GATGACCATCCGCCCAGCA	CGGACCGACAGCTGCTTGGC

### Noxious escape assays

Twenty female flies between 7–10 days old were collected and placed in single 35 mm petri dish. These flies were left for 30 minutes to acclimate to their new environment. In order to determine the noxious heat threshold, these flies were floated on a heat bath set at 42 **°**C for 55 seconds, and the noxious heat threshold was determined by the percentage of flies that climbed to the top of the petri dish during this period of time^22^. Each data point shown on Fig. 3a constitutes the mean of three technical replicates of one group of twenty female flies.

### Withdrawal latency assays

Twenty female flies between 7–10 days old were collected and placed in single 35 mm petri dish. These flies were left for 30 minutes to acclimate to their new environment. In order to determine the withdrawal latency, these flies were floated on a heat bath set at 42 **°**C and the time it took for 75% of flies to reach the top of the petri dish was measured^22^. Each data point shown on Fig. 3b constitutes the mean of three technical replicates of one group of twenty female flies.

### Statistical analyses

All statistical analyses, including student’s t-tests, two-tailed Pearson’s correlations, and Log-Rank (Mantel-Cox) test survival curve analyses were performed using GraphPad Prism 6 software.

### Statement of data availability

The datasets generated during and/or analyzed during the current study are available from the corresponding author on reasonable request.

## Electronic supplementary material

Supplementary Figures 1-5
