# Supplementary material for: Allatostatin C modulates nociception and immunity in Drosophila
Source: Sci Rep. 2018 May 14;8:7501. doi: 10.1038/s41598-018-25855-1 (PMC5951828; doi:10.1038/s41598-018-25855-1)
Supplement: Supplementary file 1 — Supplementary Figures 1-5 [file 41598_2018_25855_MOESM1_ESM.pdf]

Submitted to *Scientific Reports*

Supplementary Information for:

Allatostatin C modulates nociception and immunity in *Drosophila*

Nathaniel D. Bachtel [#1,2] , Gary A. Hovsepian[1,2], Douglas F. Nixon[2*], Ioannis Eleftherianos[1*]

[1]Department of Biological Sciences, George Washington University, Washington, DC 20052;

[2]Department of Microbiology, Immunology, and Tropical Medicine, George Washington University, Washington DC USA 20037

*Shared senior authors

#Corresponding author: Nathaniel D. Bachtel, nathaniel.bachtel@gmail.com

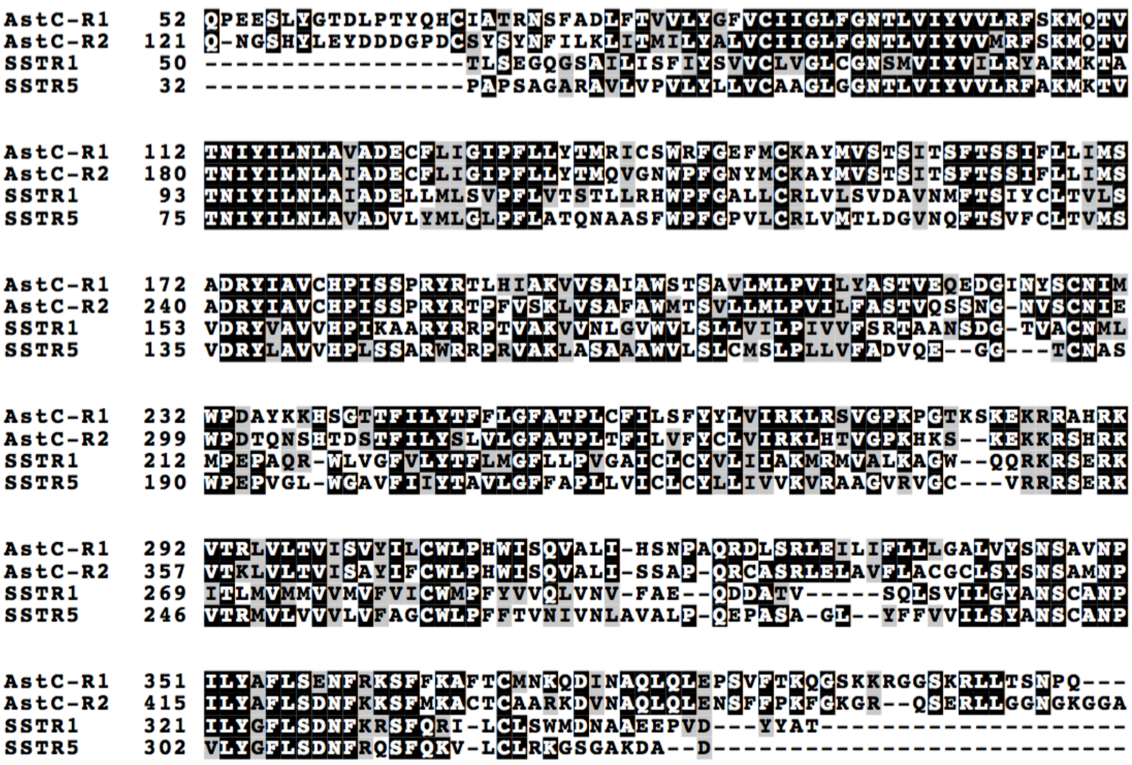

Figure S1. Multiple sequence alignment of the amino acid sequences of AstC-R1, AstC-R2, SSTR1, SSTR5. Somatostatin receptors 1 and 5, and Allatostatin C receptors 1 and 2 display strong protein sequence homology. Lack of highlighting indicates no sequence homology, grey highlighting indicates conserved amino acid residues, black highlighting indicated identical amino acid residues.

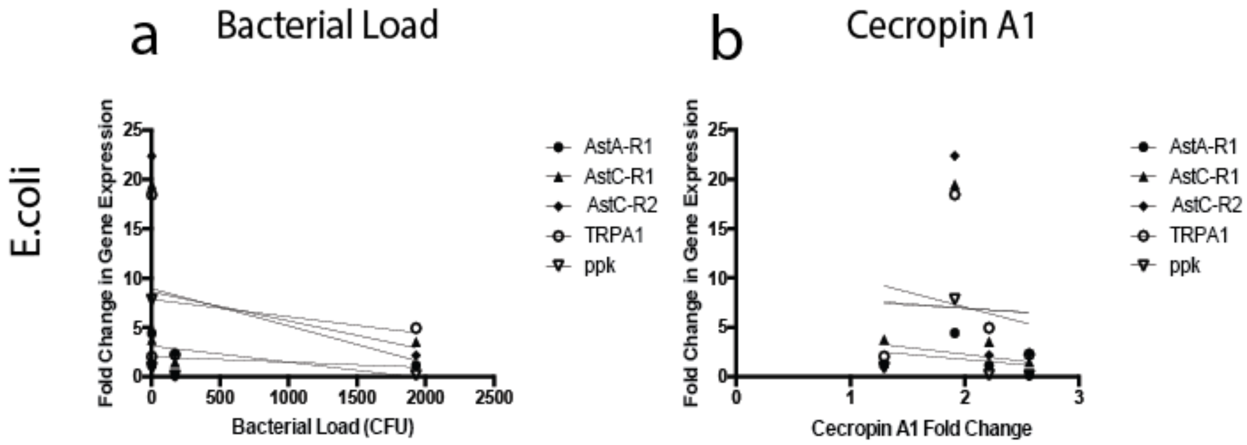

Figure S2. Nociceptive gene expression does not correlate with bacterial load or *Cecropin A1* induction upon challenge with *E. coli*. Correlation and linear regression lines for nociceptive gene expression in w^{1118} flies over time plotted against (a) bacterial load and (b) *Cecropin A1* expression following *E. coli* infection. Bacterial load does not correlate with *TRPA1*, *AstA-R1, AstC-R1*, *AstC-R2* or *ppk* gene expression upon infection with *E. coli* via two-tailed linear regression analysis. Each point plotted is the mean of 3-4 gene expression experiments using w^{1118} flies.

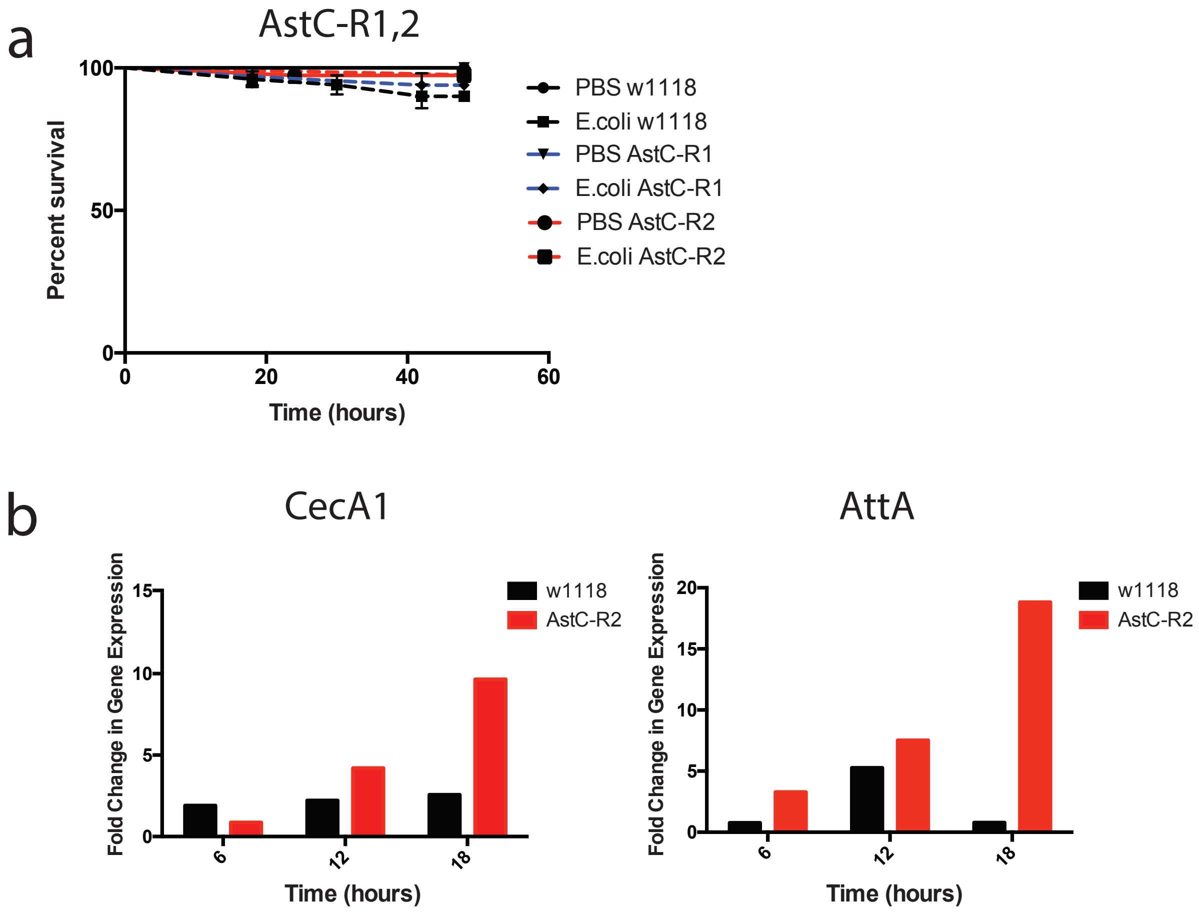

Figure S3. Survival and immune activation of *Allatostatin C-R1* and *Allatostatin C-R2* mutant flies. *AstC-R1* and *AstC-R2* RNAi knockdown mutants do not display altered survival during *E. coli* infection (n=3 groups of 20 flies for each genotype and each condition) (a). *AstC-R2* mutants do, however, display hyperactive IMD immune signaling as measured by the transcript levels of the antimicrobial peptide-encoding genes *Cecropin A1* and *Attacin A* (b). RNAi mutants were generated by crossing UAS-RNAi lines with an Actin5c Gal4 driver in order to knock the gene of interest down ubiquitously.

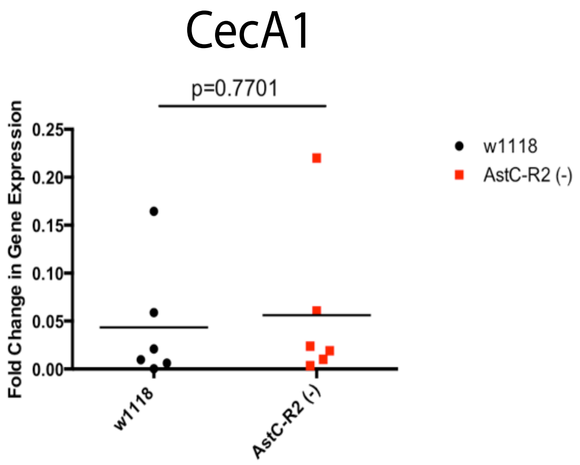

Figure S4. Immune activation in *Allatostatin C-R2* mutant flies. *AstC-R2* RNAi knockdown mutants [AstC-R2(-)] do not display hyperactive IMD signaling as measured by the transcript levels of the antimicrobial peptide-encoding gene *Cecropin A1* in the absence of bacterial challenge. RNAi mutants were generated by crossing UAS-RNAi lines with an Actin5c Gal4 driver in order to knock *AstC-R2* down ubiquitously. Differences in gene expression profiles were analyzed for statistical significance using a student's t-test (n=6 groups of 10 flies for each genotype).

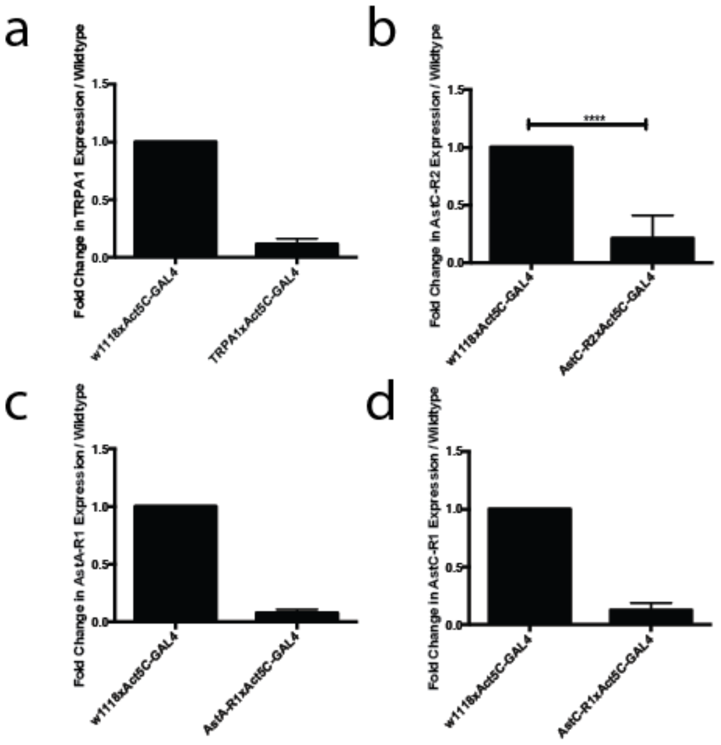

Figure S5. Validation of RNAi line knockdown efficacy. RNAi knockdown efficacy as measured by transcript levels of *TRPA1* (a), *AstC-R2* (b), *AstA-R1* (c), and *AstC-R1* (d) normalized to their background controls determine via quantitative RT-PCR. RNAi mutants were generated by crossing UAS-RNAi lines with an Actin5c Gal4 driver in order to knock the gene of interest down ubiquitously. Differences in gene expression profiles were analyzed for statistical significance using a student's t-test (n=2-3 groups of 10 flies for each genotype, ****=p<0.0001)
